# Combining body mass index and waist height ratio to assess the relationship between obesity and serum uric acid levels in adolescents

**DOI:** 10.3389/fped.2023.1176897

**Published:** 2023-05-18

**Authors:** Shan Liu, Wei Wei, Yuan Cheng, Jing-Yi Chen, Yang Liu, Zhi-Ping Wu, Meng-Die Hu, Heng Zhao, Xiao-Feng Li, Xin Chen

**Affiliations:** ^1^ Department of Epidemiology, School of Public Health, Dalian Medical University, Dalian, China; ^2^ Department of Neurosurgery, Central Hospital of Dalian University of Technology, Dalian, China; ^3^ Institute of Health Science, China Medical University, Shenyang, China

**Keywords:** serum uric acid, obesity, adolescent, body mass index, waist height ratio

## Abstract

**Background:**

The study aims to explore the relationship between obesity and serum uric acid in adolescents by combining body mass index and waist height ratio.

**Methods:**

475 adolescents in our study were classified as normal weight without central obesity (NW), normal weight but central obesity (NWCO), overweight or obesity without central obesity (OB) and overweight or obesity with central obesity (OBCO). Odds ratios (OR) and 95% confidence intervals (CI) for hyperuricemia were calculated using a logistic regression model. The dose-response association between obesity indicators and serum uric acid were explored by restricted cubic spline model.

**Results:**

The highest serum uric acid level and the OR for hyperuricemia were found in the OBCO group, regardless of sex. After controlling for waist height ratio, the risk of hyperuricemia increased with increasing body mass index in boys and girls. The restricted cubic spline model showed that boys had higher ORs for hyperuricemia at the 25th and 75th percentiles of body mass index than for waist height ratio and girls had a higher OR for hyperuricemia than waist height ratio at the 25th percentile of body mass index.

**Conclusions:**

Hyperuricemia in adolescence was not only associated with the overweight or obesity in BMI, but with the combination of overweight or obesity in BMI and central obesity in WHtR. However, in boys and girls, the increased risk of hyperuricemia associated with elevated body mass index was significantly better than that of waist height ratio.

## Introduction

Serum uric acid (SUA) is an endogenous and dietary soluble metabolic end product, and after free filtration by the glomerulus, about 90% of filtered uric acid (UA) is reabsorbed at the renal tubules (1). Hyperuricemia is a metabolic disorder caused by a disorder of purine metabolism that results in excessive production and/or decreased excretion of UA (2). In addition to gout, SUA is also a clinical marker of other diseases, including hypertension, chronic kidney disease, hypertriglyceridemia, obesity, atherosclerotic heart disease and metabolic syndrome (MetS) (3, 4). A meta-analysis conducted among Chinese adults showed that from 2000 to 2014, the prevalence of hyperuricemia in mainland China was 13.3% (5). However, the prevalence of hyperuricemia in Chinese children and adolescents was not well characterized. A recent meta-analysis of children and adolescents showed that the total prevalence of hyperuricemia was estimated to be 23.3%, with 26.6% of boys and 19.8% of girls (6). During childhood and adolescence, SUA levels gradually increase from early childhood to physical development and plateau by age 15–17 (7). Hyperuricemia in adolescence is often thought to be associated with insulin resistance (IR) (8), MetS (3), and hypertension (4). Studies had shown that obesity, especially central obesity, increases xanthine oxidase activity in adipose tissue and leads to higher SUA production and lower SUA renal clearance (9), and the prevalence of hyperuricemia in adolescents with obesity was several times higher than in the general adolescents (10).

Body mass index (BMI) remains the most widely used indicator for assessing obesity in children and adults, but BMI does not reflect central obesity (11). It cannot separate muscle mass from bone and fat mass (12). Waist height ratio (WHtR) has been considered as an easily measured anthropometric indicator for detecting central obesity and assessing the relationship between cardiometabolic risk factor variables and central intraperitoneal obesity (13, 14). The results of a study conducted in Chinese adults have shown that WHtR is an independent and better predictor of hyperuricemia than BMI and WC (15). However, no study results have shown that WHtR is a stronger predictor of hyperuricemia than the other two indicators in children and adolescents. And, it has been suggested that single consideration of one of BMI, WC, WHtR provides limited information on fat distribution (16). Studies conducted in adults combined BMI and WHtR to study hyperuricemia in subjects with different body weights and different central obesity, thus taking into account both the weight and fat distribution of the subjects (17).

The aims of our study were to evaluate SUA levels in students aged 14–17 years and to explore the relationship between obesity and SUA in adolescents by combining BMI and WHtR.

## Methods

### Participants

This cross-sectional study was based on school and conducted in a high school randomly selected from Huanggu District, Shenyang City, Liaoning Province. A total of 481 14–17 years old high school students were included in this study by cluster random sampling initially, of whom 475 students (194 boys and 281 girls) remained in the sample for analysis in the current work after eliminating students with missing physical measurements and biochemical data records.

### Anthropometric evaluation

Anthropometric measurement was carried out by trained professionals following the standard protocol. Referring to the equipment and methods of GB/T26343, fully automatic electronic scales were used to record anthropometric data. We uniformed and calibrated the instruments we used.

Participants were required to wear light clothes when WC (cm), height (cm) and weight (kg) were measured. A portable height measuring device (model TZG, Jiangyin Hongya Science and Education Equipment Co., Ltd.) was used to measure height with an accuracy of 0.1 cm. When measuring weight, the students stood in the central of the scale. Recorded the reading in “kg” to one decimal place. BMI was calculated as body weight (kg) divided by the square of height (m^2^). WC was measured with a tape measure at the midpoint between the iliac crest and lowest rib with 0.1 cm precision when the student was standing, with adequate exposure of the abdomen and spontaneous breathing. WHtR was calculated as WC (cm) divided by the height (cm). Blood pressure was measured using an auscultatory mercury sphygmomanometer (model XJ1ID, China) with a suitable cuff for children. Participants rested in a seated position for at least 5 min before having their blood pressure measured. The cuff was placed approximately 2 cm above the crease of the elbow on the students' right arm. Each student was measured 3 consecutive times with two intervals of no less than 30 s. The measurements were averaged after removing abnormal values and recorded in mmHg.

### Biochemical measurements

Biochemical measurement analysis was performed by the Laboratory Department of Shengjing Hospital, China Medical University. SUA was measured using photoelectric colorimetry (Hitachi 7,600 clinical analyzer, Hitachi, Tokyo, Japan). Fasting plasma glucose (FPG), total cholesterol (TC), high-density lipoprotein (HDL), low density lipoprotein (LDL) and triglycerides (TG), were measured by using clinical methods (Hitachi 7,600 clinical analyzer, Hitachi, Tokyo, Japan). Automated particle-enhanced turbidimetric immunoassays were performed of cystatin C (CYSC) using an Architect I16200 automatic analyzer (Architect, Shandong, China). Furthermore, all the students' blood samples were taken early in the morning on an empty stomach.

### Questionnaire survey

The questionnaire was required to be completed by the students and their parents in face-to-face interviews with well-trained personnel, and it was used to collect information about the population such as name, age, gender, ethnicity and address.

The Ethics Committee of Dalian Medical University gave its approval to these studies. We obtained informed consent from all young people and their parents.

### Definitions of obesity and hyperuricemia

According to the BMI standards advocated by China for children and adolescents aged 3–18 years old by age and gender, students were classified as normal, overweight and obesity (18). In addition, the presence and absence of central obesity were defined as WHtR ≥0.48 and <0.48 (18). Based on BMI and central obesity status, students in this study were classified as normal weight without central obesity (NW), normal weight but central obesity (NWCO), overweight or obesity without central obesity (OB) and overweight or obesity with central obesity (OBCO).

Based on previous scholarly research on hyperuricemia in children and adolescents (19), in our present study, hyperuricemia was defined as SUA concentrations exceeding the normal range for age and sex: 111–353 µmol/L in children aged 14–15 years; 143–339 µmol/L for girls >15 years old and 202–416 µmol/L for boys >15 years old.

### Statistical methods

For the description of study population characteristics: continuous variables were expressed as means and standard deviations (SD) or medians and interquartile ranges, and categorical variables were expressed as numbers and percentages. Students were analyzed stratified by sex. Comparisons were performed using *t*-tests (two-tailed), non-parametric statistical tests and chi-square tests for the two gender groups. We identified factors independently associated with hyperuricemia by univariate logistic regression. A trend test for the different types of obesity and the risk of hyperuricemia was also performed, using NW as reference, resulting in odds ratios (OR) and 95% confidence intervals (CI). We then further controlled for BMI or HWtR, using binary logistic regression to calculate ORs and 95% CIs for other types of obesity using a trend test for hyperuricemia in students with non-centric obesity or normal weight as a reference. To further explore the measured response relationships between SUA and BMI and between SUA and WHtR, we used a restricted cubic spline (RCS) model which nodes at the 10th, 50th and 90th percentiles to fit the linear regression model and the logistic regression model. In order to control the influence factor of age, we also converted BMI into BMI *Z*-score according to the BMI growth standards of WHO (20). In exploring the risk of hyperuricemia, we used the median of BMI and BMI *Z*-score and the WHtR cut-off value of 0.48 for boys and girls as reference values, combined with a binary logistic regression model to plot dose-response curves.

We performed two-sided statistical tests on all data and differences were considered statistically significant when the *P*-value was less than 0.05. SPSS 21.0 was used for comparison between groups and logistic regression analysis, and R (version 3.6.3) was used for RCS model.

## Results

### Baseline characteristics of study participants

Tables 1, 2 summarized the baseline characteristics of all boys and girls with different types of obesity. A total of 475 students aged 14–17 years (194 boys and 281 girls) were included in our analysis. For the four different obesity groups, the NW group accounted for the highest proportion in both boys and girls (51.55%; 51.60%) and the OB group accounted for the lowest proportion in both boys and girls (8.25%; 6.76%). Supplementary Table S1 compared the baseline characteristics between different genders. There was no significant difference in BMI levels between boys and girls (21.69; 21.56 kg/m^2^, respectively, *P* > 0.05), and no difference in WHtR between them (0.47; 0.46, respectively, *P* > 0.05). Boys had signiﬁcantly higher SUA levels than girls, with a mean level of 428.12 ± 75.14 μmol/L in boys and 322.20 ± 60.50 µmol/L in girls (*P* < 0.05).

**Table 1:** Characteristics of boys with different types of obesity.

	Obesity types				*P*
	NW	NWCO	OB	OBCO	
*N*	100 (51.55)	21 (10.82)	16 (8.25)	57 (29.38)	
Age (years)	16.00 (15.00–16.00)	16.00 (16.00–17.00)	16.00 (15.25–17.00)	16.00 (16.00–16.00)	0.015
Height (cm)	173.73 ± 6.44	170.21 ± 6.38	175.65 ± 6.44	171.63 ± 6.10	0.014
Weight (kg)	61.10 (56.53–65.73)	61.90 (54.80–66.40)	76.80 (73.23–80.70)	81.30 (74.10–91.25)	0.000
BMI (kg/m^2^)	20.14 ± 1.70	21.09 ± 1.27	24.77 ± 1.40	28.45 ± 3.85	0.000
WC (cm)	73.00 (70.00–77.00)	85.00 (82.50–87.50)	80.00 (78.25–83.00)	91.00 (86.00–99.50)	0.000
WHtR	0.42 (0.41–0.44)	0.50 (0.49–0.51)	0.46 (0.44–0.47)	0.52 (0.50–0.57)	0.000
SBP (mmHg)	125.00 (117.00–135.75)	121.00 (120.00–133.00)	128.50 (120.25–140.75)	139.00 (125.50–150.50)	0.000
DBP (mmHg)	69.50 (64.00–74.00)	67.00 (55.50–73.50)	70.00 (61.25–75.25)	72.00 (64.50–79.00)	0.214
Hypertension	35 (35.00)	7 (33.33)	6 (37.50)	40 (70.18)	0.000
CYSC (mg/L)	0.97 ± 0.10	0.96 ± 0.11	0.97 ± 0.06	0.97 ± 0.12	0.891
TC (mmol/L)	3.47 (3.13–3.90)	3.45 (3.14–3.69)	3.41 (3.08–4.06)	3.73 (3.30–4.20)	0.089
TG (mmol/L)	0.63 (0.51–0.85)	0.70 (0.56–0.91)	0.82 (0.54–0.99)	0.91 (0.60–1.20)	0.011
HDL (mmol/L)	1.26 (1.11–1.48)	1.15 (1.02–1.40)	1.14 (0.95–1.36)	1.13 (0.97–1.26)	0.005
LDL (mmol/L)	1.92 (1.55–2.09)	1.81 (1.69–2.14)	1.84 (1.56–2.51)	2.22 (1.75–2.60)	0.007
FPG (mmol/L)	4.71 (4.45–5.00)	4.38 (4.21–4.62)	4.58 (4.34–4.85)	4.68 (4.43–4.96)	0.007

Values are mean ± SD, *n* (%), or median (interquartile range).

BMI, body mass index; NW, normal weight without central obesity; NWCO, normal weight with central obesity; OB, obesity without central obesity; OBCO, obesity with central obesity; WC, waist circumference; WHtR, waist-to-height ratio; TG, triglyceride; HDL, high density lipoprotein; LDL, low density lipoprotein; FPG, fasting plasma glucose; CYSC, cystatin C; SBP, systolic blood pressure; DBP, diastolic blood pressure; TC, total cholesterol; SUA, serum uric acid.

**Table 2:** Characteristics of girls with different types of obesity.

	Obesity types				*P*
	NW	NWCO	OB	OBCO	
N	145 (51.60)	59 (21.00)	19 (6.76)	58 (20.64)	
Age (years)	16.00 (15.00–16.00)	16.00 (16.00–17.00)	15.00 (15.00–16.00)	16.00 (15.00–16.00)	0.000
Height (cm)	163.03 ± 5.65	159.30 ± 6.73	162.22 ± 4.93	160.90 ± 5.34	0.000
Weight (kg)	53.50 (49.20–58.00)	53.30 (50.80–57.70)	63.50 (60.40–67.40)	68.55 (63.08–75.53)	0.000
BMI (kg/m^2^)	20.22 (18.74–21.55)	21.50 (20.35–22.69)	24.22 (23.70–24.37)	26.36 (24.82–28.63)	0.000
WC (cm)	70.00 (67.00–74.00)	82.00 (79.00–85.00)	72.00 (70.00–77.00)	87.00 (80.00–92.00)	0.000
WHtR	0.43 (0.41–0.45)	0.51 (0.49–0.53)	0.45 (0.44–0.46)	0.54 (0.50–0.56)	0.000
SBP (mmHg)	112.00 (105.00–120.00)	115.00 (109.00–125.00)	120.00 (112.00–127.00)	122.00 (116.00–132.50)	0.000
DBP (mmHg)	68.12 ± 9.72	68.05 ± 12.85	68.26 ± 8.14	69.95 ± 11.29	0.714
Hypertension	30 (20.69)	17 (28.81)	8 (42.11)	28 (48.26)	0.001
CYSC (mg/L)	0.84 (0.79–0.91)	0.85 (0.79–0.93)	0.87 (0.84–0.95)	0.88 (0.83–0.95)	0.048
TC (mmol/L)	3.94 (3.62–4.39)	3.72 (3.28–4.44)	4.14 (3.56–4.86)	3.92 (3.49–4.56)	0.076
TG (mmol/L)	0.79 (0.63–1.02)	0.72 (0.53–1.02)	0.80 (0.62–1.13)	0.90 (0.71–1.39)	0.016
HDL (mmol/L)	1.51 (1.30–1.76)	1.33 (1.22–1.54)	1.50 (1.25–1.77)	1.26 (1.09–1.51)	0.000
LDL (mmol/L)	2.14 (1.80–2.40)	2.07 (1.62–2.49)	2.26 (1.93–2.65)	2.22 (1.83–2.73)	0.098
FPG (mmol/L)	4.50 (4.31–4.78)	4.48 (4.25–4.58)	4.58 (4.25–4.74)	4.43 (4.30–4.77)	0.205

Values are mean ± SD, *n* (%), or median (interquartile range).

BMI, body mass index; NW, normal weight without central obesity; NWCO, normal weight with central obesity; OB, obesity without central obesity; OBCO, obesity with central obesity; WC, waist circumference; WHtR, waist-to-height ratio; TG, triglyceride; HDL, high density lipoprotein; LDL, low density lipoprotein; FPG, fasting plasma glucose; CYSC, cystatin C; SBP, systolic blood pressure; DBP, diastolic blood pressure; TC, total cholesterol; SUA, serum uric acid.

### SUA levels in boys and girls based on different combinations of BMI with WHtR

**Figure 1:**
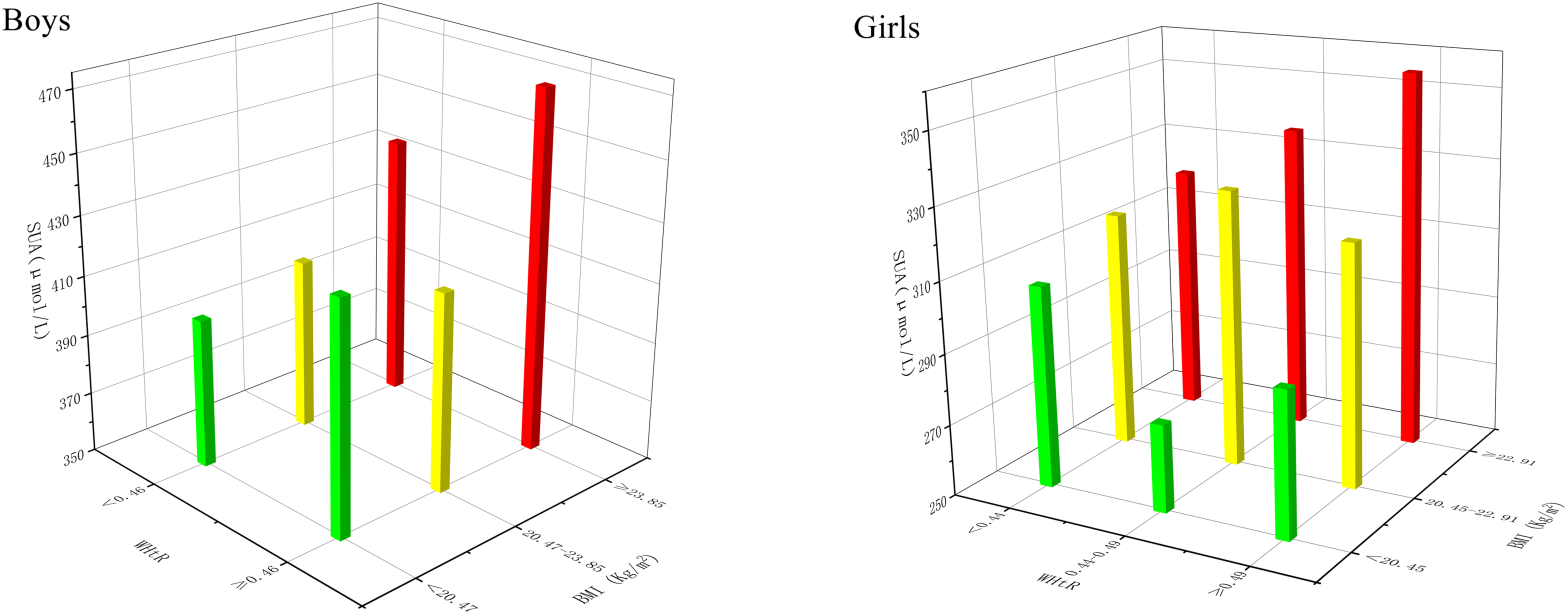
SUA in boys and girls on different combinations of BMI and WHtR. SUA, serum uric acid; BMI, body mass index. WHtR, waist-to-height Ratio; Red: the highest level of BMI; Yellow: the middle level of BMI; Green: the lowest level of BM.

Table 3 showed the SUA levels of different types of obesity in girls and boys. Through comparison, it was found that the highest SUA level in boys and girls was in OBCO group (466.16; 359.79 µmol/L), while the lowest SUA level was in NWCO group (403.57; 310.42 µmol/L). Significant differences between groups occurred only when the NWCO group was compared with the OBCO group, and when the NW group was compared with the OBCO group (*P* < 0.05). In addition, as showed in Figure 1, we grouped the BMI of boys in tertiles, the WHtR in median, but girls' BMI and WHtR in their respective tertiles. In this grouping case, SUA levels were also the highest when both BMI and WHtR were at their highest levels, and SUA consistently increased with BMI in girls, regardless of WHtR.

**Table 3:** SUA levels in boys and girls with different types of obesity.

Types	Total		Boys		Girls	
	*N*	SUA	*N*	SUA	*N*	SUA
NW	245	351.57 ± 78.18	100	410.77 ± 66.72	145	310.74 ± 56.43
NWCO	80	334.88 ± 73.22	21	403.57 ± 72.28	59	310.42 ± 56.43
OB	35	378.00 ± 81.40	16	433.25 ± 71.88	19	331.47 ± 56.67
OBCO	115	412.51 ± 87.84	57	466.16 ± 78.06	58	359.79 ± 61.15
* *	*P*		*P*		*P*	
NW*NWCO	0.093		0.659		0.971	
OB*OBCO	0.040		0.134		0.079	
NW*OB	0.064		0.218		0.134	
NWCO*OBCO	0.000		0.002		0.000	
NW*OBCO	0.000		0.000		0.000	

Values are mean ± SD.

NW, normal weight without central obesity, NWCO, normal weight with central obesity; OB, obesity without central obesity; OBCO, obesity with central obesity; SUA, serum uric acid.

### Blood biochemical indicators associated with hyperuricemia

We identified factors independently associated with hyperuricemia by univariate logistic regression. In boys, Age, TC and CYSC were factors associated with hyperuricemia. At the same time, in girls, SBP, TC, TG, LDL and CYSC were factors associated with hyperuricemia (*P* < 0.1) (Table 4).

**Table 4:** Blood biochemical indicators associated with hyperuricemia.

Variable		Boys			Girls	
	OR	95%CI	*P*	OR	95%CI	*P*
Age	0.33	(0.21–0.54)	0.000	1.14	(0.82–1.59)	0.442
SBP	1.01	(0.99–1.03)	0.406	1.03	(1.01–1.05)	0.008
DBP	0.99	(0.97–1.02)	0.541	1.01	(0.98–1.03)	0.600
TC	1.49	(0.93–2.39)	0.097	1.60	(1.10–2.33)	0.014
TG	1.57	(0.80–3.11)	0.191	2.91	(1.57–5.38)	0.001
HDL	1.06	(0.55–2.02)	0.867	0.90	(0.40–2.00)	0.794
LDL	1.51	(0.87–2.64)	0.139	1.98	(1.25–3.15)	0.004
FPG	0.91	(0.67–1.23)	0.529	0.76	(0.36–1.60)	0.464
CYSC	28.86	(1.57–530.49)	0.024	664.89	(28.85–15,325.56)	0.000

OR, odds ratio; 95% CI, 95% conﬁdence interval; TG, triglyceride; HDL, high density lipoprotein; LDL, low density lipoprotein; FPG, fasting plasma glucose; CYSC, cystatin C; SBP, systolic blood pressure; DBP, diastolic blood pressure; TC, total cholesterol.

### OR and 95% CI for hyperuricemia of different types of obesity in boys and girls

**Figure 2:**
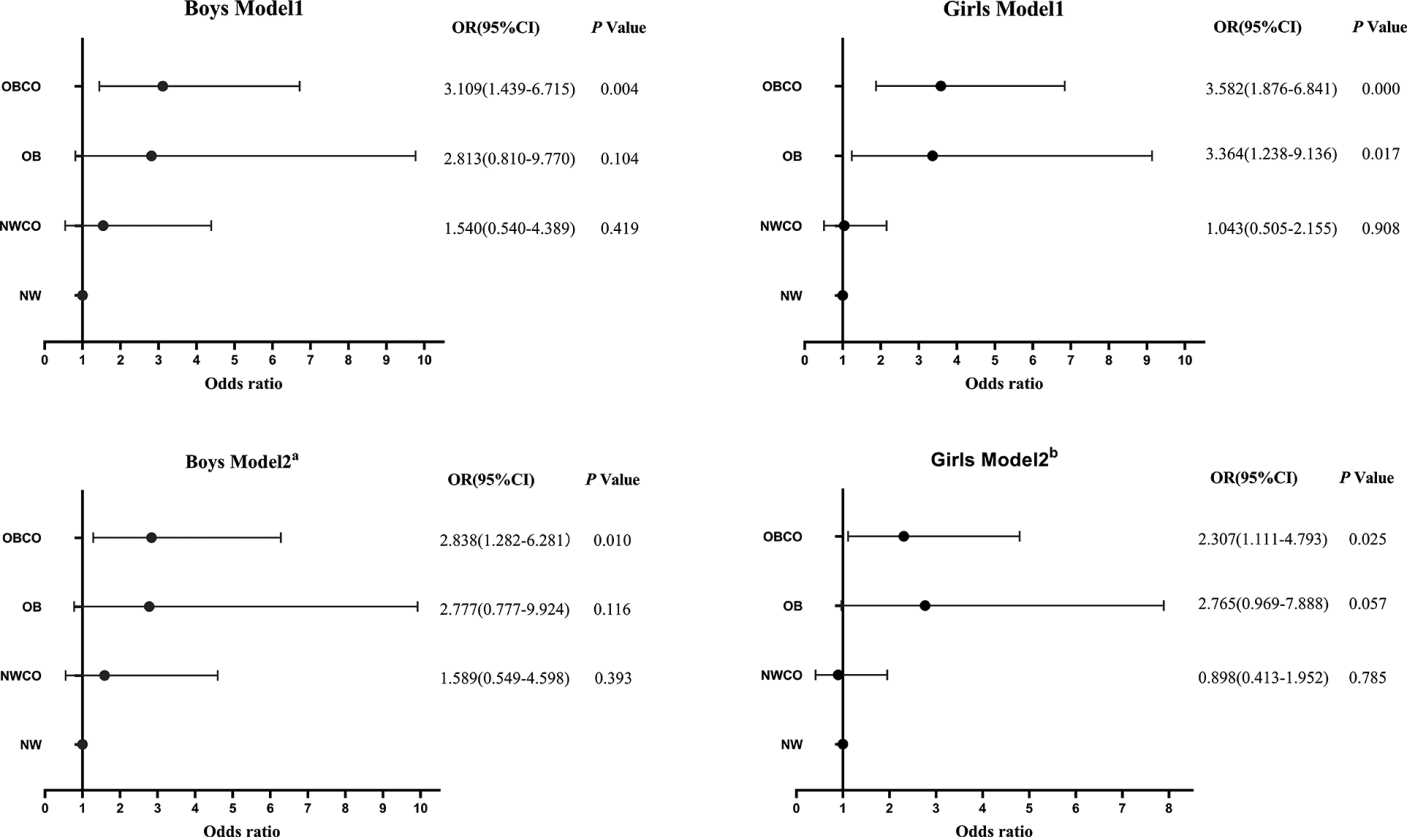
OR and 95% CI for hyperuricemia in different obesity types. Model 1: adjusted for age. Model 2a: adjusted for model 1 + TC + CYSC. Model 2b: adjusted for model 1+ TG + TC + LDL + CYSC and SBP. OR, odds ratio; 95% CI, 95% conﬁdence interval, NW, normal weight without central obesity; NWCO, normal weight with central obesity; OB, obesity without central obesity OBCO, obesity with central obesity.

As shown in Figure 2, the risk of hyperuricemia in NWCO, OB and OBCO increased sequentially compared with NW in boys. In model 2, the risk of hyperuricemia was increased 59%-fold in the NWCO group compared to NW (OR = 1.59, 95% CI: 0.55–4.60, *P* = 0.393), and nearly 3-fold in both the OB and OBCO groups compared to NW (OR = 2.78, 95% CI: 0.78–9.92, *P* = 0.116; OR = 2.83, 95% CI: 1.28–6.28, *P* = 0.010). However, this trend is significant only for the OBCO group (*P* < 0.05).

In girls, Model 1 was similar to that of boys, and the risk of hyperuricemia in the NWCO, OB, and OBCO groups was sequentially increased compared to NW. However, when adjusted factors were added, in Model 2, the highest risk of hyperuricemia compared with NW was observed in the OB group (OR = 2.77, 95% CI: 0.97–7.89, *P* = 0.057), followed by OBCO group (OR = 2.31, 95% CI: 1.11–4.79, *P* = 0.025), and finally NWCO group (OR = 0.90, 95% CI: 0.41–1.95, *P* = 0.79).

### OR and 95% CI for hyperuricemia in boys and girls after control of BMI or WHtR

In Table 5, We controlled BMI to study the effects of WHtR on hyperuricemia. When students had severe central obesity, regardless of sex and BMI, the risk of hyperuricemia was increased compared with non-central obesity. But none of these trends were statistically significant (*P* > 0.05).

**Table 5:** Or and 95% CI of hyperuricemia after control BMI and WHtR.

	Boys				Girls			
	*N*	hyperuricemia	OR (95%CI)	*P*	*N*	hyperuricemia	OR (95%CI)	*P*
NW or NWCO								
Non-Central obesity	100	56 (56.00)	1.00	—	145	33 (22.76)	1.00	—
Central obesity	13	8 (61.54)	1.85 (0.52–6.59)	0.345	20	2 (10.00)	0.42 (0.09–1.95)	0.269
Severe Central obesity	8	3 (37.50)	1.08 (0.21–5.64)	0.932	39	13 (33.33)	1.42 (0.60–3.35)	0.430
OB or OBCO								
Non-Central obesity	16	11 (68.75)	1.00	—	19	9 (47.37)	1.00	—
Central obesity	11	8 (72.73)	0.68 (0.09–5.31)	0.716	15	7 (46.67)	0.91 (0.21–3.90)	0.899
Severe Central obesity	46	35 (76.09)	1.08 (0.23–4.98)	0.927	43	23 (53.49)	1.29 (0.39–4.24)	0.679
NW or OB								
Normal weight	100	56 (56.00)	1.00	—	144	32 (22.22)	1.00	—
Overweight	15	10 (66.67)	2.73 (0.76–9.73)	0.123	17	7 (41.18)	2.48 (0.80–7.67)	0.115
Obesity	—	—	—	—	1	1 (100)	—	—
NWCO or OBCO								
Normal weight	21	11 (52.38)	1.00	—	60	16 (26.67)	1.00	—
Overweight	25	17 (68.00)	1.02 (0.24–4.39)	0.981	37	15 (40.54)	1.57 (0.61–4.05)	0.347
Obesity	33	27 (81.82)	2.41 (0.54–10.72)	0.247	22	16(72.73)	6.44(1.70–24.35)	0.006

After controlling for WHtR, the risk of hyperuricemia increased progressively with increasing BMI in both boys and girls, regardless of WHtR. It was worth noting that among girls with abnormal WHtR, the risk of hyperuricemia was nearly 6.5 times higher in obesity girls than in those with normal BMI, and this risk was significant (OR = 6.44, 95% CI:1.70–24.35, *P* = 0.006).

### Dose-response analysis of the association between SUA and obesity indicators

**Figure 3:**
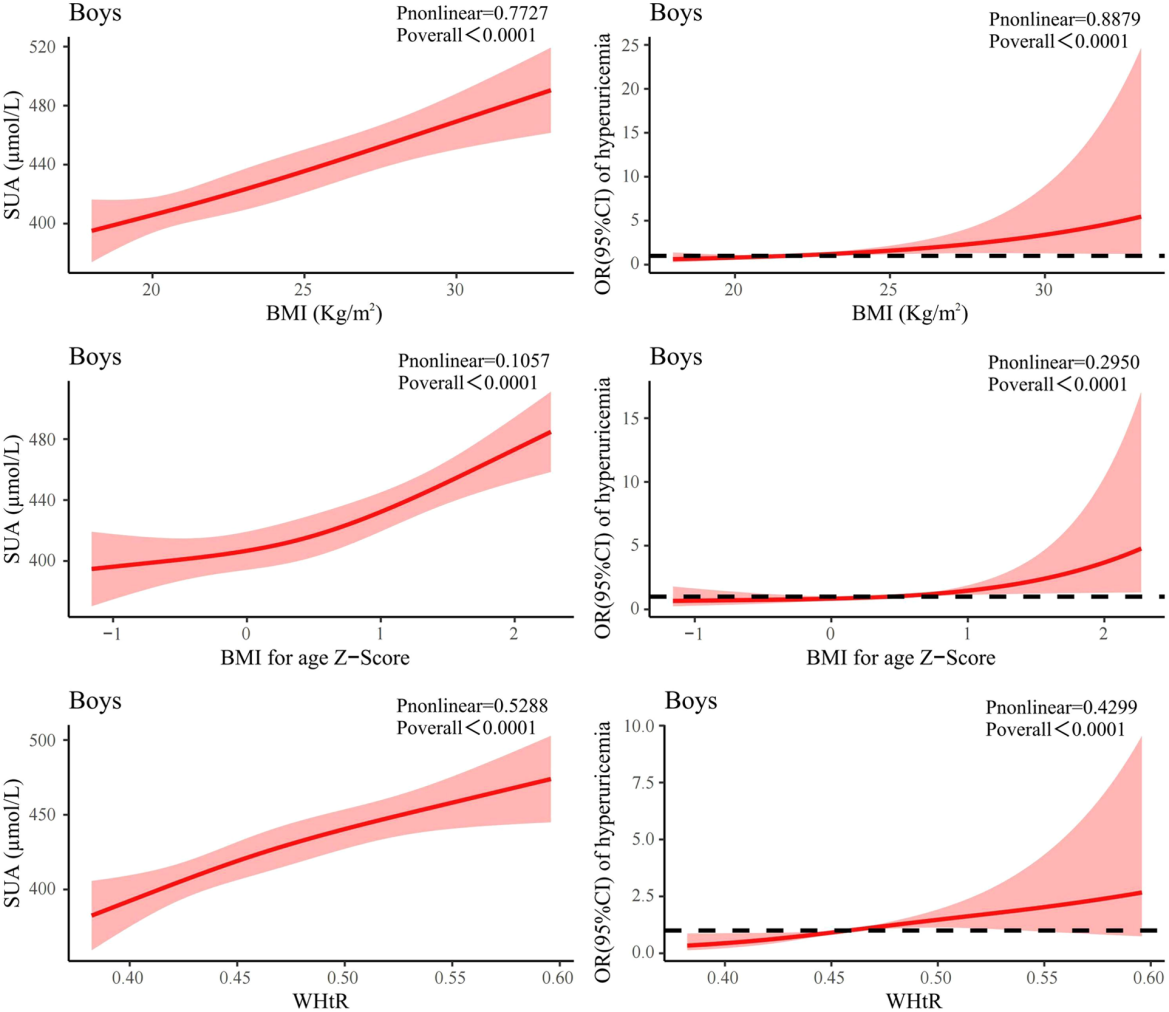
Association between obesity indicators and SUA and association between obesity indicators and risk of hyperuricemia using RCS regression model in boys. SUA, serum uric acid; OR, odds ratio; RCS, restricted cubic spline; BMI, body mass index; WHtR, waist-to-height Ratio.

**Figure 5:**
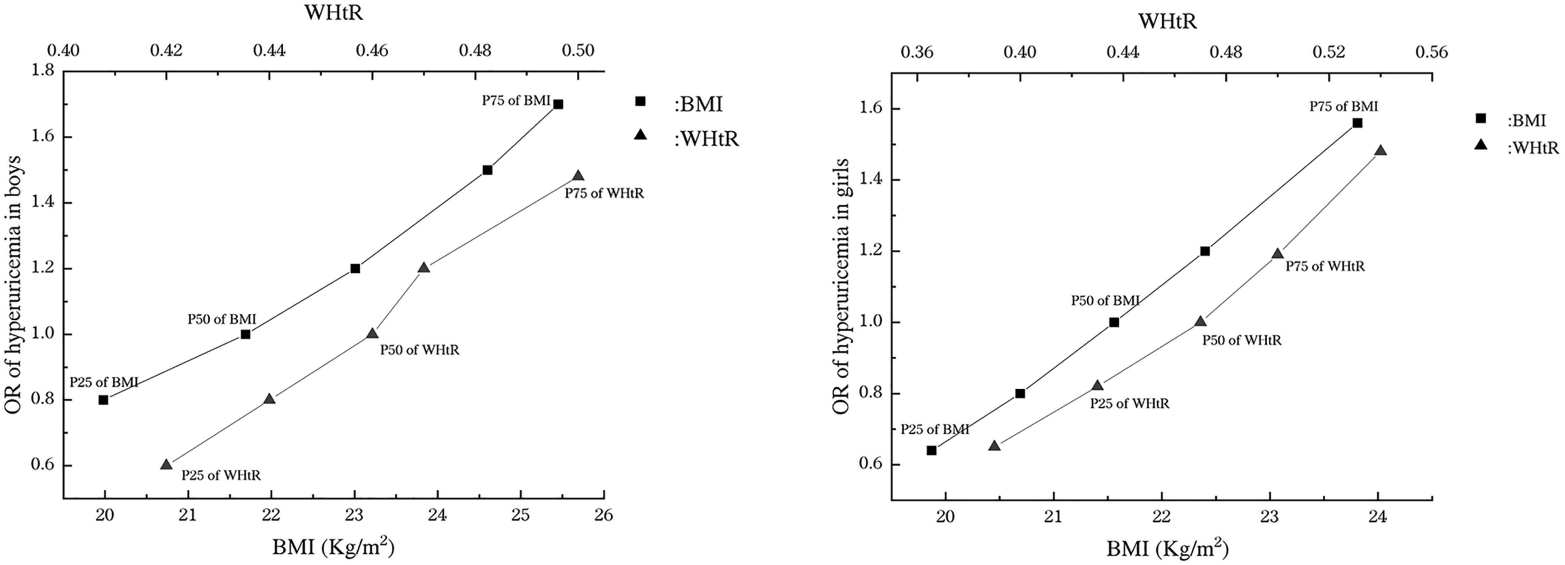
Association of BMI and WHtR with the risk of hyperuricemia in boys and girls. OR, odds ratio; BMI, body mass index; WHtR, waist-to-height Ratio; P25, the 25th percentile; P50, the 50th percentile; P75, the 75th percentile.

The dose-response association between obesity indicators (BMI, WHtR) and SUA was explored by RCS. As shown in Figure 3, SUA levels and the risk of hyperuricemia in boys were positively correlated with BMI (*P* nonlinear = 0.773, *P* nonlinear =0.888), BMI Z-score (*P* nonlinear = 0.106, *P* nonlinear = 0.295) and WHtR (*P* nonlinear = 0.529, *P* nonlinear = 0.430). In addition, when boys reached the 25th and 75th percentiles of BMI (19.98 kg/m^2^, 25.45 kg/m^2^), the OR for hyperuricemia were 0.80 (95% CI: 0.58–1.11) and 1.70(95% CI: 1.21–2.39). Besides, when WHtR reached the 25th and 75th percentile (0.42, 0.50), the OR were only 0.60 (95% CI: 0.40–0.90) and 1.48(95% CI: 1.13–1.94). The ORs for the 25th quartile and 75th quartile of BMI were all higher than the ORs for the 25th quartile and 75th quartile of WHtR (Figure 5).

**Figure 4:**
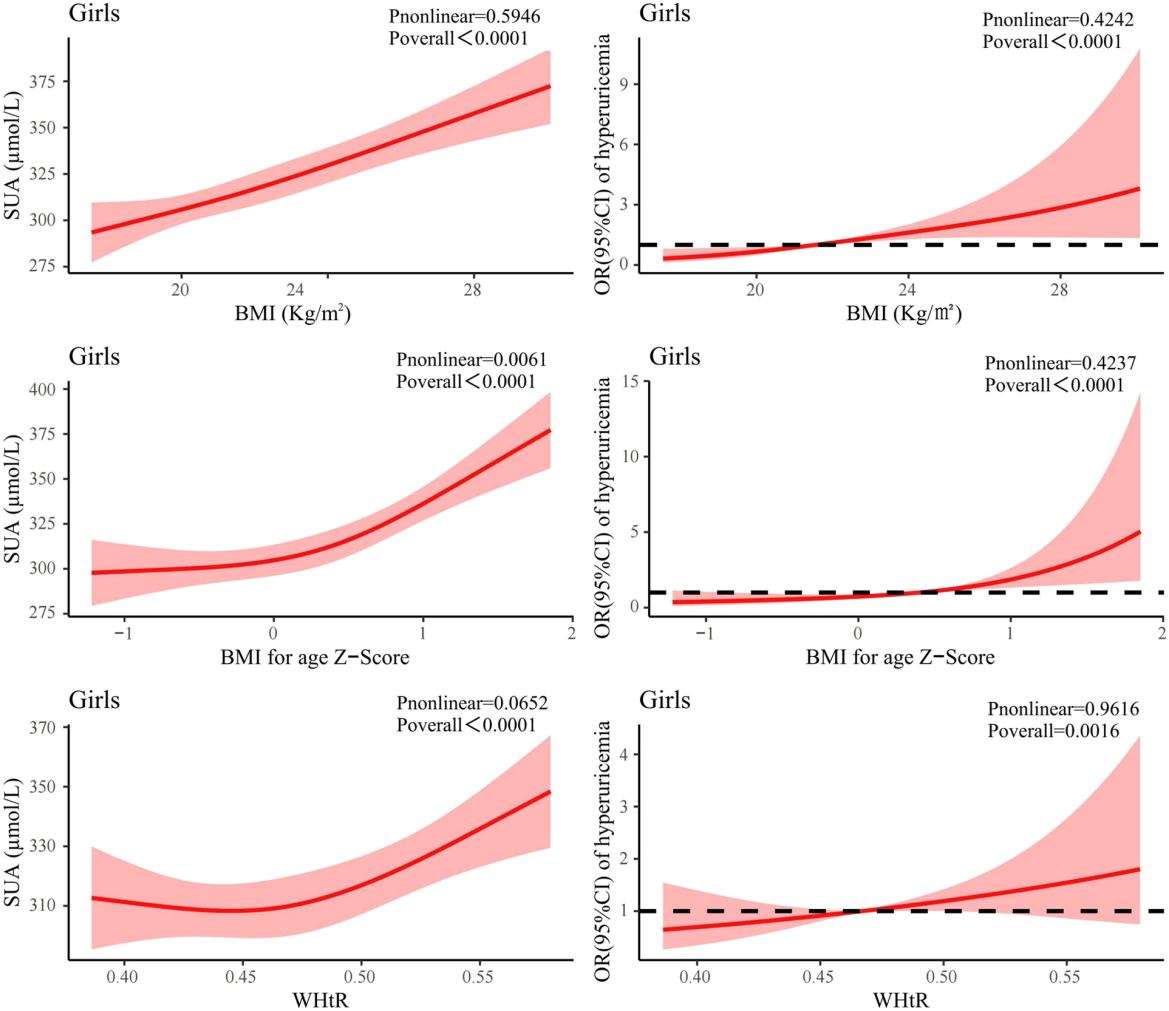
Association between obesity indicators and SUA and association between obesity indicators and risk of hyperuricemia using RCS regression model in girls. SUA, serum uric acid; OR, odds ratio; RCS, restricted cubic spline; BMI, body mass index; WHtR, waist-to-height Ratio.

As shown in Figure 4, girls' BMI was similar to that of boys, with SUA level and the risk of hyperuricemia positively associated with BMI (*P* nonlinear = 0.595, *P* nonlinear = 0.424). There was also a positive association between BMI *Z*-score and SUA level in girls, but it was non-linear (*P* nonlinear = 0.0061). However, the association between WHtR and SUA level in girls was J-like shaped on a continuous scale with SUA reaching the lowest point at a WHtR of 0.44 (*P* nonlinear = 0.065). In addition, when girls reached the 25th and 75th percentiles of BMI (19.87 kg/m^2^, 23.81 kg/m^2^), the OR for hyperuricemia were 0.65 (95% CI: 0.47–0.90) and 1.54 (95% CI: 1.25–1.89). Moreover, when WHtR reached the 25th and 75th percentile (0.43, 0.50), the OR were 0.82 (95% CI: 0.59–1.13) and 1.20 (95% CI: 1.00–1.42). The OR for 25th quartile of BMI was lower than the OR for 25th quartile of WHtR, while the OR for 75th quartile of BMI was higher than the OR for 75th quartile of WHtR (Figure 5).

## Discussion

This present study proposed that hyperuricemia in adolescence was not only associated with the overweight or obesity in BMI, but with the combination of overweight or obesity in BMI and central obesity in WHtR. In addition, we also found that the increased risk of hyperuricemia associated with elevated BMI was significantly better than that of WHtR in both boys and girls. SUA levels and the risk of hyperuricemia were all positively correlated with BMI and WHtR in boys. In girls, SUA levels and the risk of hyperuricemia were positively associated with BMI. However, the association between WHtR and SUA levels in girls on a continuous scale was J-like shaped, and as WHtR increased, SUA levels showed a trend of decreasing and then increasing.

In our study, boys and girls were 14–17 years old. The Tanner stage is used to divide the pubertal development internationally (21). Tanner 1 is the stage where secondary sexual characteristics do not appear, which is pre-puberty; Tanner 2∼3 is the stage when secondary sexual characteristics appear and sexual organs begin to develop, which is the early stage of puberty. Tanner 4–5 is the stage of rapid development of sexual organs and sexual maturity, which is the middle and late stage of puberty (22). A previous muti-center cross-sectional study showed that the median age and 95% CI of Chinese girls assessed by breasts and pubic hair at Tanner 4 were 14.21 (13.94–14.49) and 15.29 (14.99–15.62). Moreover, the median age and 95% CI of Chinese girls assessed by breasts and pubic hair at Tanner 5 were 17.39 (16.97–17.91) and 18.34 (17.88–18.91) (23). In addition, the cross-sectional study showed that the median age and 95% CI of Chinese boys assessed by genital, pubic hair and testicular volume at Tanner 4 were 14.39 (14.12–14.67), 14.76 (14.59–14.94) and 13.02 (12.84–13.21). Moreover, the median age and 95% CI of Chinese boys assessed by genital, pubic hair and testicular volume at Tanner 5 were 16.17 (15.93–16.45), 16.48 (16.24–16.75) and 15.78 (15.33–16.32) (24). Therefore, it can be considered that our subjects (14–17 years old) were in Tanner 4 and Tanner 5, which is the middle and late stage of puberty.

Our study found that boys' SUA levels were higher than girls', which is the same as other studies of children and adolescents (25). The reason for the gender difference in SUA between boys and girls during adolescence is that the SUA of boys increase sharply, while the SUA of girls remain relatively unchanged (26). Studies have shown that increased renal urate retention may be the main underlying mechanism of increased SUA (27). Adolescents have a progressive decline in renal fractional UA excretion and clearance, leading to increased UA retention (28). However, estrogen in girls can increase UA clearance, promote UA excretion, and reduce SUA levels (29), and estradiol, on the other hand, can inhibit isolated xanthine oxidase (UA-producing enzyme), and can also pharmacologically reduces circulating UA (30). In our study, girls aged 14–17 were in the pubertal developmental stage and had already secreted sex hormones in their bodies, so they had less UA retention and lower levels of SUA than boys. Another factor that contributes to higher SUA levels in boys than in girls was male testosterone. Testosterone has been reported to stimulate isolated xanthine oxidase to pharmacologically increase circulating UA, suggesting that testosterone may contribute to sex differences in UA (30, 31). Apart from this, some studies have found that higher SUA in adolescent boys is associated with lower sex hormone binding globulin (SHBG). The reason for this may be that SHBG can bind to testosterone, thereby reducing the level of free and bioavailable testosterone (26). It could also be due to some unknown mechanism. Considering the specificity of growth and development of adolescent children and the differences in the distribution of SUA between boys and girls, we conducted a stratified analysis by gender in the analysis.

Causes of elevated SUA or hyperuricemia in children and adolescents are similar to those in adults, however, secondary hyperuricemia due to other conditions is more common in adolescents, such as cyanotic congenital heart disease, kidney disease (32). Besides, elevated SUA or hyperuricemia in children and adolescents is also closely related to lifestyle factors such as obesity (33). Some scholars analyzed the carotid intima-media thickness (IMT), SUA, creatinine and serum triglyceride of 120 obese children and 50 healthy controls, and found that the concentration of SUA in children with obesity was higher than that in the healthy group significantly (34). This relationship between obesity and SUA levels was also shown in our study. This finding is similar to those of studies in Italy (35), Thailand (25) and Brazil (36). A recent follow-up study of children and adolescents aged 4–18 also found that 65 children who lost weight during follow-up had reduced SUA levels, while 23 children who gained weight during follow-up had increased SUA levels (37). Furthermore, Rocha EPAA and others suggested that SUA could be used to distinguish between metabolically healthy status and metabolically unhealthy status in children and adolescents with overweight and obesity, and that the higher the level of SUA, the higher the risk of metabolically unhealthy (38). Elevated SUA levels were associated with obesity, possibly due to IR (33). Gil-Campos and others studied the relationship between SUA and IR syndrome in children and concluded that elevated SUA levels in children with obesity during prepubertal compared to lean children appear to be an early metabolic change associated with other features of IR syndrome (39). The reason for this was that IR due to obesity reduced UA excretion by the kidneys over the proximal renal tubules, leading to hyperuricemia (40). In addition to IR, leptin was found to be a causative factor for hyperuricemia in obese patients and was hypothesized to be an association between the two (41). Leptin regulated fat mass and body weight by suppressing food intake and stimulating energy expenditure, and numerous adult studies have shown that leptin is a biomarker for obesity, IR and MetS (42). However, there have been no studies in children and adolescents to demonstrate an association between leptin and hyperuricemia. In addition, differences in SUA levels between subjects with normal BMI and subjects with overweight/obesity may be related to oxidative stress. UA is generally considered to be an important anti-oxidant in human body and it is responsible for 55% of the antioxidant scavenging of extracellular free radicals (43). However, from a biological perspective, UA is not only anti-oxidant, but also pro-oxidation (44). Extracellular UA acts primarily as an anti-oxidant, but pro-oxidation also occurs when UA levels are too high. At this point, the extracellular UA reacts with myeloperoxidase to form a compound with pro-oxidation properties called hydroperoxide urate, which plays a pro-oxidation role (45). Oxidative stress occurs when there is an imbalance between the production of reactive oxygen species (ROS) and cellular anti-oxidant factors (46). Supraphysiological production of ROS is one of the major determinants of overall obesity-related health deterioration, and oxidative stress has also been shown to be a significant feature of obesity (47). Therefore, this may be one of the reasons for the differences in SUA levels and prevalence of hyperuricemia between adolescents with overweight/obesity and adolescents with normal weight.

In the current study, BMI and WHtR were combined and the results showed that students had the highest SUA levels when both BMI overweight or obesity and central obesity as defined by WHtR were present. However, when BMI and WHtR were controlled separately, the increased risk of hyperuricemia due to increased BMI was more pronounced. This may indicate that there is a stronger association between BMI and hyperuricemia compared with the association between WHtR and hyperuricemia in adolescents. This is similar to the results of a longitudinal analysis on children and adolescents of APV registries in Germany/Austria/Switzerland (48) and a study of adults conducted in Liaoning province, China (16). However, a previous study of adults conducted in China indicated that WHtR is an independent and better predictor of hyperuricemia than BMI and WC (15). This phenomenon can be explained in two aspects. Firstly, leptin was found to be a causative factor for hyperuricemia in obese patients (41). Previous studies also showed a high correlation between leptin levels and BMI in both females and males and concluded that leptin production is proportional to adipose tissue mass (49). Another study also demonstrated a high association between leptin levels and overall adipose tissue depots rather than with fat depot in a certain body part (50). Secondly, as an indicator of central obesity, WHtR mainly reflects the accumulation of abdominal fat. However, adolescents are at a special stage of rapid physical growth and development, fat distribution and body shape may be changing rapidly. Therefore, the overall degree of obesity and the degree of obesity accumulation reflected by BMI may be more strongly associated with hyperuricemia in adolescence. This characteristic of adolescents in our study may also account for the lower SUA levels in the NWCO group than in the NW group. Compared with adolescents in the NW group, adolescents in the NWCO group only had more abdominal fat, but the general body weight was not higher and this may lead that the SUA level of adolescents in NWCO group not higher than that of the adolescents in NW group. In fact, NWCO is a new type of obesity that combined general obesity and central obesity, and has been used more in adult studies than in children and adolescents. As far as we know, our study is the first to use this type of obesity (NWCO) to assess the association between obesity and hyperuricemia in children and adolescents. Therefore, more studies in children and adolescents are needed to be conducted to explore the relationship between NWCO and SUA levels and the relationship between different indicators of obesity and hyperuricemia.

There were some other interesting findings in our study. Results of univariate Logistic regression in our study also showed that SBP, TG and LDL was associated with hyperuricemia in girls but not in boys. This phenomenon was consistent with a study of adolescents in America (51) and a study of adults using NHANES data from 2009 to 2018 (52). Potential mechanisms include the influence of sex hormones and genetic differences between the sexes (53, 54). Studies have shown a positive and independent correlation between xanthine oxidase (XO) levels and hypertension (55). XO is a UA-producing enzyme, and estradiol in women can inhibit isolated XO (30), resulting in a stronger association of SBP with hyperuricemia in girls. Regarding differences in the relationship between lipid profile and hyperuricemia in boys and girls, it has been suggested that estrogen can promote UA excretion and regulate lipid metabolism in the kidney (56). Therefore, reducing blood pressure and lipid profile levels in girls should be considered to control hyperuricemia.

A J-like shape association between WHtR and SUA in girls was found in our study, with a decreasing SUA before WHtR reached 0.44 and then increasing. However, this trend was not found among boys. Since the age distribution of our subjects was during puberty (14–17 years), this interesting difference may be due to the different levels of pubertal development in boys and girls. However, no other study found this J-like shape association between WHtR and girls' SUA. Thus, prospective studies with a large sample size should to be carried out to verify the differences between WHtR and SUA levels in boys and girls. According to the guidelines, adult hyperuricemia was defined as men whose SUA ≥ 416 μmol / L and women whose SUA ≥ 357 µmol/L (57). However, SUA levels change during development in children and adolescents. Thus, age—and sex-related SUA reference values should be taken into account when defining hyperuricemia in children and adolescents. In Masaru Kubota's report (58), two previous UA reference values for children and adolescents of different genders and ages have been summarized. In addition, for Chinese children and adolescents, the normal range of UA level was defined as 119–416 µmol/L, and hyperuricemia was defined as UA level > 416 µmol/L, according to *Zhufutang Practical Pediatrics* (59). Considering that the students in our study were 14–17 years old, we used the criteria in a previous study (19). The future need for separate reference values for hyperuricemia in adolescents remains to be explored.

There were several limitations in this analysis. First, the adolescents in our study were obtained by cluster random sampling in Shenyang, Liaoning Province, and were not nationally representative. Second, in the limited data available, our analysis did not take into account key negative factors such as family history, dietary status and IR levels. Therefore, further population-based prospective studies should be conducted to conquer these limitations and to investigate systematically the relationship between obesity and SUA and hyperuricemia in boys and girls during adolescence.

In conclusion, hyperuricemia in adolescence was not only associated with the overweight or obesity in BMI, but with the combination of overweight or obesity in BMI and central obesity in WHtR. However, in both boys and girls, the increased risk of hyperuricemia associated with elevated BMI was significantly better than that of WHtR. Prospective studies with larger sample sizes should be conducted to assess SUA levels in adolescents of different genders with different BMI and WHtR.

## Data Availability

The raw data supporting the conclusions of this article will be made available by the authors, without undue reservation.
