# Supplementary material for: Combining body mass index and waist height ratio to assess the relationship between obesity and serum uric acid levels in adolescents
Source: Front Pediatr. 2023 May 18;11:1176897. doi: 10.3389/fped.2023.1176897 (PMC10232991; doi:10.3389/fped.2023.1176897)
Supplement: Supplementary file 1 [file Table1.docx]

**Supplementary Table 1.** Study sample characteristics of subjects according to sex.

|  | Boys | Girls | *P* |
| --- | --- | --- | --- |
| N | 194 | 281 | —— |
| Age (years) | 16.00(15.00-16.00) | 16.00(15.00-16.00) | 0.272 |
| Height (cm) | 172.89±6.46 | 161.75±5.96 | 0.000 |
| Weight (kg) | 66.00(59.85-77.45) | 56.50(51.10-62.95) | 0.000 |
| BMI (kg/m^2^) | 21.69(19.98-25.45) | 21.56(19.87-23.81) | 0.108 |
| WC (cm) | 79.00(73.00-87.00) | 75.00(70.00-81.50) | 0.000 |
| WHtR | 0.46(0.42-0.50) | 0.47(0.43-0.50) | 0.332 |
| Obesity types |  |  |  |
| NW | 100(51.55) | 145(51.60) | 0.012 |
| NWCO | 21(10.82) | 59(21.00) |  |
| OB | 16(8.25) | 19(6.76) |  |
| OBCO | 57(29.38) | 58(20.64) |  |
| SBP (mmHg) | 128.00(120.00-141.00) | 116.00(108.00-124.00) | 0.000 |
| DBP (mmHg) | 70.00(63.75-75.00) | 68.00(62.00-74.50) | 0.183 |
| Hypertension | 88(45.36) | 83(29.54) | 0.001 |
| SUA (μmol/L) | 428.12±75.14 | 322.20±60.50 | 0.000 |
| Hyperuricemia | 121(62.37) | 87(30.96) | 0.000 |
| CYSC (mg/L) | 0.97±0.10 | 0.86±0.09 | 0.107 |
| TC (mmol/L) | 3.54(3.15-3.96) | 3.91(3.52-4.46) | 0.000 |
| TG (mmol/L) | 0.71(0.54-1.02) | 0.80(0.62-1.09) | 0.002 |
| HDL (mmol/L) | 1.21(1.05-1.38) | 1.45(1.22-1.68) | 0.000 |
| LDL (mmol/L) | 1.94(1.67-2.36) | 2.15(1.79-2.49) | 0.000 |
| FPG (mmol/L) | 4.63(4.40-4.96) | 4.49(4.28-4.72) | 0.000 |

Values are mean±SD, n (%), or median (interquartile range).

BMI body mass index, NW normal weight without central obesity, NWCO normal weight with central obesity, OB obesity without central obesity, OBCO obesity with central obesity, WC waist circumference, WHtR waist-to-height ratio, TG triglyceride, HDL high density lipoprotein, LDL low density lipoprotein, FPG fasting plasma glucose, CYSC cystatin C, SBP systolic blood pressure, DBP diastolic blood pressure, TC total cholesterol, SUA serum uric acid.
